# Modified double dumbbell-shaped split-ring resonator-based negative permittivity metamaterial for satellite communications with high effective medium ratio

**DOI:** 10.1038/s41598-021-98703-4

**Published:** 2021-09-29

**Authors:** Md Bellal Hossain, Mohammad Rashed Iqbal Faruque, Sikder Sunbeam Islam, Mohammad Tariqul Islam

**Affiliations:** 1 Space Science Centre (ANGKASA), Institute of Climate Change (IPI), Universiti Kebangsaan Malaysia, 43600 UKM Bangi, Selangor D.E. Malaysia; 2 Department of Electrical and Electronic Engineering, Noakhali Science and Technology University, Noakhali, Bangladesh; 3 Department of Electrical and Electronic Engineering, International Islamic University Chittagong, Chittagong, Bangladesh; 4 Department of Electrical, Electronic and Systems Engineering, Universiti Kebangsaan Malaysia, 43600 UKM Bangi, Selangor D.E. Malaysia

**Keywords:** Electrical and electronic engineering, Characterization and analytical techniques

## Abstract

Metamaterial with negative permittivity demonstrate excellent performance in cutting-edge technology. Thus, this study modified the double dumbbell-shaped split-ring resonator (MDD-SRR) based negative permittivity for satellite communications. The proposed MDD-SRR unit cell comprises a square-shaped split-ring resonator and two dumbbell-shaped rings. Some parts of the outer square ring were extended to enlarge the electrical length which altered the inductance of the metamaterial unit cell. The dimension of the proposed unit cell is 9 × 9 × 1.524 mm^3^, fabricated on a Rogers RT6002 (lossy) substrate material. Based on the results, five resonances for the transmission coefficient were achieved at frequencies of 2.896 GHz, 8.11 GHz, 9.76 GHz, 12.48 GHz and 13.49 GHz, including the S, X and Ku band satellite communication frequency bands through numerical simulation in a high-frequency electromagnetic simulator Computer Simulation Technology (CST) microwave studio. Negative permittivity at frequencies ranging from 2.896–3.76 GHz, 8.11–8.592 GHz, 9.76–10.784 GHz, 12.496–12.768 GHz, 13.504–14.4 GHz, were observed and extracted using the Robust and Nicolson–Ross–Weir (NRW) methods. Meanwhile, an effective medium ratio (EMR) measured at 11.51 to 2.896 GHz specified the goodness of the metamaterial unit cell for satellite communication with higher bandwidth and gain. The simulated, circuit model and measured results that were compared for validation purposes indicated that the simulation results, the equivalent circuit model results and measured results occupied each other. Moreover, the numerical simulation of the double dumbbell-shaped metamaterial unit cell was performed using a High-Frequency Structure Simulator (HFSS) to confirm the results. To evaluate the parametric study, the proposed unit cell was subjected to change different substrate types, change of split gap of rings, change of direction of electromagnetic field propagation, and structural optimization. In conclusion, the S, X and Ku-bands in the proposed metamaterial are competent for satellite communications as they are also investigated using an array of a unit cell.

## Introduction

In the year 1968, Victor Vesselago^1^ introduced the idea of a non-existent material, where it has a negative permittivity (ε) and permeability (µ) in a certain frequency span. However, no one expected this idea to become a state of the art technology until 1999. In 2000, Smith et al.^2^ described the double negative material referred to as left-handed metamaterial indicating negative effective medium parameters. Metamaterials can be incorporated into a vast array of applications due to their exotic electromagnetic properties. The applications include bandwidth enhancement^3,4^, biomedical applications^5^, superlenses^6^, invisibility cloaks^7^, specific absorption rate (SAR) reduction^8,9^, obstacle sensing^10^, chemical detection^11^, electromagnetic absorber^12–14^, energy harvesting^15,16^, wavefront manipulation^17,18^, polarisation conversion^19^, vortex beam generators^20,21^ etc. Having said that, ordinary materials are not relevant for these applications, where only metamaterials can be utilised due to their peculiar properties. Ordinary materials only generate positive permittivity and permeability which are commonly found in nature called a double positive media (DPS). However, some materials found in nature do exhibit negative permittivity and permeability occasionally but not simultaneously are called a single negative media (SNG). Meanwhile, metamaterials that generate negative ε and µ are not found in nature but are physically realisable by incorporating geometrical shape structures on a dielectric substrate, called a double negative media (DNG). Having said that, double negative and single negative metamaterials are both being applied for groundbreaking discoveries. An earlier study demonstrated a man-made magnetic material based on an SRR, in which the SRR was printed on a dielectric substrate with several shapes like as circular, rectangular, hexagonal, etc. Now, different types of shapes such as the English alphabet, engineering and religious symbol, Greek letters and letter-like symbols are used as a shape of SRR fabricated on dielectric medium.

Reference^11^ introduced a G-shaped resonator-based metamaterial absorber to detect various types of oils and liquefied chemicals in the X-band region. A different type of metamaterial designed with an eSRR resonator was proposed in Ref.^10^, where it was used for obstacle sensing in the C-band of the microwave region. Because of the widespread applications in wireless communications, imaging and radar applications, the manipulation of electromagnetic (EM) waves became highly desirable due to their effective control. Meanwhile, the proposed wideband three-layered chiral structure metasurface can simultaneously combine polarisation and wavefront of the transmitted wave^17^. A novel concentric ring-based crossed line SNG metamaterial structure indicated by Azeez et al.^22^ was fabricated on the most commonly used FR-4 dielectric substrate operating in Ku- and Ka-bands with an EMR of 4.44. A. F. Almutairi et al.^23^ introduced a DNG metamaterial with 5.5 × 5.5 mm^2^ dimension, which was validated using an 18 × 20 array structure. The DNG metamaterial was incorporated into C-band microwave applications with an EMR value of 8. Another study presented an epsilon-negative metamaterial for single-layer rectangular invisible cloaking activity that covers the C-band with low EMR^24^. While a separate study proposed a hexagonal-shaped split ring resonator with a gap coupled for the microwave absorptance approach^25^. This resonator was fabricated using an FR-4 dielectric substrate material covering C and X frequency bands with a size of 10 × 10 mm^2^ at an EMR of 8.4. Whereas a modified circular electric resonator-based metamaterial indicated a double negative characteristic, with a frequency band of 9.7–10.5 GHz and 15–15.7 GHz with an EMR of 5.45 at 5 × 5 mm^2^^26^. In another study^27^, a horizontally inverted double L-shaped metamaterial was used for microwave applications covering the C-, X- and Ku-bands with a dimension of 10 × 10 mm^2^. The results indicated a double negative approach to the resonance frequencies. Meanwhile^28^ investigated the use of Jerusalem cross-unit cell-based near-zero index (NZI) metamaterial to amplify the high antenna gain operating at 43 GHz.

Besides that, a new novel metamaterial presented in Ref.^29^ was manufactured on a Flame retardant-4 dielectric substrate. The metamaterial displayed ENG properties in S-band, showing the DNG property whether Rogers RT 6010 dielectric substrate is utilized. Whereas^30^ demonstrated a split-ring resonator-based metamaterial in the form of a double H-shape to exhibit quadruple resonance for satellite applications. This metamaterial structure yielded an EMR value of 10.75 at a size of 9 × 9 mm^2^. Moreover, Ramachandran et al.^31^ investigated a metamaterial with a composite circular-shaped resonator with ENG properties for C-band and DNG characteristics for Ku-band. In another study^32^, a negative permittivity metamaterial based on a cross-coupled interlinked resonator was implemented for satellite and radar approaches. This metamaterial unit cell was validated through the advanced design system (ADS) software and was fabricated using different arrays. The unit cell size and EMR were 8 × 8 mm^2^ and 8.03, respectively. Additionally, a polarisation-dependent tunnelled metamaterial unit cell was proposed to improve the field characteristics^33^. The EMR of this proposed metamaterial was 3.39 with a dimension of 10 × 8 mm^2^. On the other hand, a Greek-key pattern-based multiband metamaterial was investigated for miniaturisation which was also validated through the 10 × 10 array of the metamaterial unit cell using the free-space measurement technique^34^. The numerical size of the pattern and EMR were 10 × 10 mm^2^ and 12.5, respectively. In Ref.^35^, a metamaterial-based absorber was proposed covering frequency bands of 1.8 GHz, 2.45 GHz and 5.80 GHz for microwave images. Thus, a metamaterial-based energy harvester was designed for satellite communication and a global system for mobile communications at frequencies, 0.9, 1.37, 1.61 and 2.55 GHz^36^. A new M-band nested U-ring resonator was proposed for the X- and Ku-bands^37^, in which the resonant frequency can be independently managed by changing the arm length. In another study^38^, the proposed DNG metamaterial with a compact square shape ring was successfully developed for quintuple resonance frequencies to extend the C, X and Ku frequency bands for satellite applications with good EMR.

The choice of substrate material is important for a metamaterial unit cell design because it affects the resonance frequency and EM properties. Subsequent studies were organized on different types of substrate materials. In 2018, a typical TD-shaped metamaterial unit cell based on the flexible NiAl2O4 substrate was designed for triple-band applications^39^. This study utilised the frequency range of 4–18 GHz by numerically mapping the metamaterial structure in CST and HFSS simulator. A new metamaterial unit cell was designed and analysed on a variety of substrates such as FR-4, Rogers RT 6010, lossy polyimide and aluminium nitride substrate materials^40^. The unit exhibited DNG properties for Rogers RT 6010 and lossy polyimide substrate materials, whereas the other substrate materials demonstrated SNG properties. However, all resonant frequencies covered the S-band.

In this study, a modified double dumbbell-shaped split-ring resonator (MDD-SRR) based negative permittivity metamaterial was developed using Rogers RT-6002 dielectric substrate material with quintuple resonance frequencies and high EMR indicating S-, X- and Ku-bands for satellite communications. In satellite and radar systems, the S-, X- and Ku-bands have excellent applications. Particularly, the S-band is used from 2 to 4 GHz to design invisible cloaks in military applications, while X- and Ku-bands ranging from 8 to 12 GHz and 12 to 18 GHz, respectively, can be used for satellite communications. Moreover, various parameters for the proposed metamaterial such as ε, µ and η were calculated using the Robust and Nicolson–Ross–Weir (NRW) techniques. The behaviour of the electromagnetic field analysis is also examined. A circuit model similar to the proposed metamaterial unit cell was also outlined and the optimised values of the lumped components were calculated using the ADS software. The high EMR value assessed the goodness of fit of the metamaterial unit cell. Different array types, including 1 × 2, 2 × 2 and 8 × 8 arrays is also analysed. The S-parameter results of the unit cell were simulated using the HFSS. Finally, the modified double dumbbell-shaped split-ring resonator-based metamaterial is fabricated and measured to validate the simulation results, which is suitable for satellite applications. The proposed unit cell and 1 × 2 array structure were fabricated and measured using Agilent N5227 PNA Microwave Network Analyzer showing an excellent agreement with the simulated results.

### Unit cell design

**Figure 1:**
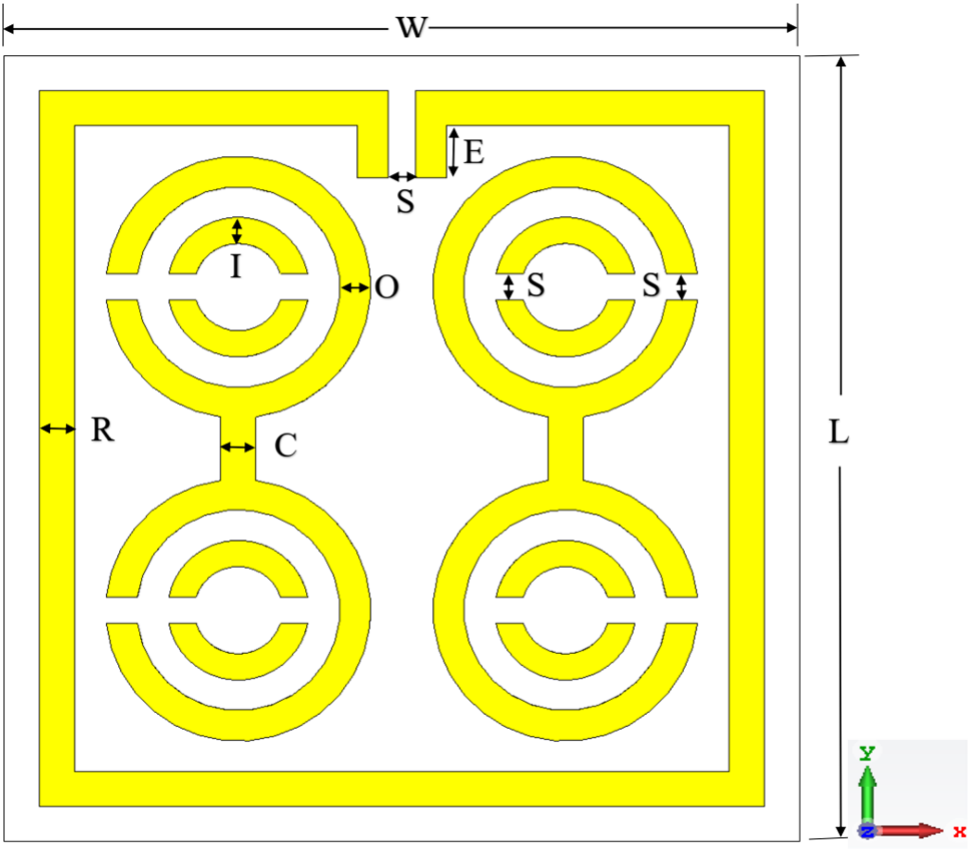
Perspective view of the proposed MDD-SRR unit cell.

The proposed MDD-SRR was built on the Rogers RT6002 substrate as presented in Fig. 1. The proposed MDD-SRR unit cell structure contains four concentric circular metallic rings forming a dumbbell shape, etched on the Rogers dielectric substrate. Rogers RT6002 dielectric materials play an important role in the performance and electrical properties including low dielectric and electrical signal loss, low outgassing for satellite communication, etc. The dumbbell shape was formed by two interconnected double SRRs with 0.2 mm splits at opposite ends has two splits in the inner ring. Meanwhile, some parts of the outer square split ring were extended to expand the electrical length. The dielectric constant of the Rogers RT6002 substrate material was 2.94, where the loss tangent was 0.0012. Having said that, the thickness of the substrate and copper metallic structure was 1.524 mm and 0.035 mm, respectively. The whole split gap of the proposed unit cell was 0.2 mm based on the trial and error to maintain uniformity that offers a maximum number of resonances with excellent EMR. Whereby, the CST simulator was used to perform numerical simulations of the unit cell. Table 1 illustrates all the split gap dimensions, metal length along with the inner and outer ring radius.

**Table 1:** The specification parameters of the proposed MDD-SRR unit cell structure.

Parameter	Dimensions
Thickness of the outer square ring, R	0.4 mm
Thickness of the outer circular ring, O	0.35 mm
Thickness of the inner circular ring, I	0.3 mm
Length and Width of the substrate, L, W	9 mm
Width of connector, C	0.4 mm
The gap between the whole strip, S	0.2 mm
Length of extended part of outer square ring, E	0.6 mm

### Effective medium parameters extraction method

**Figure 2:**
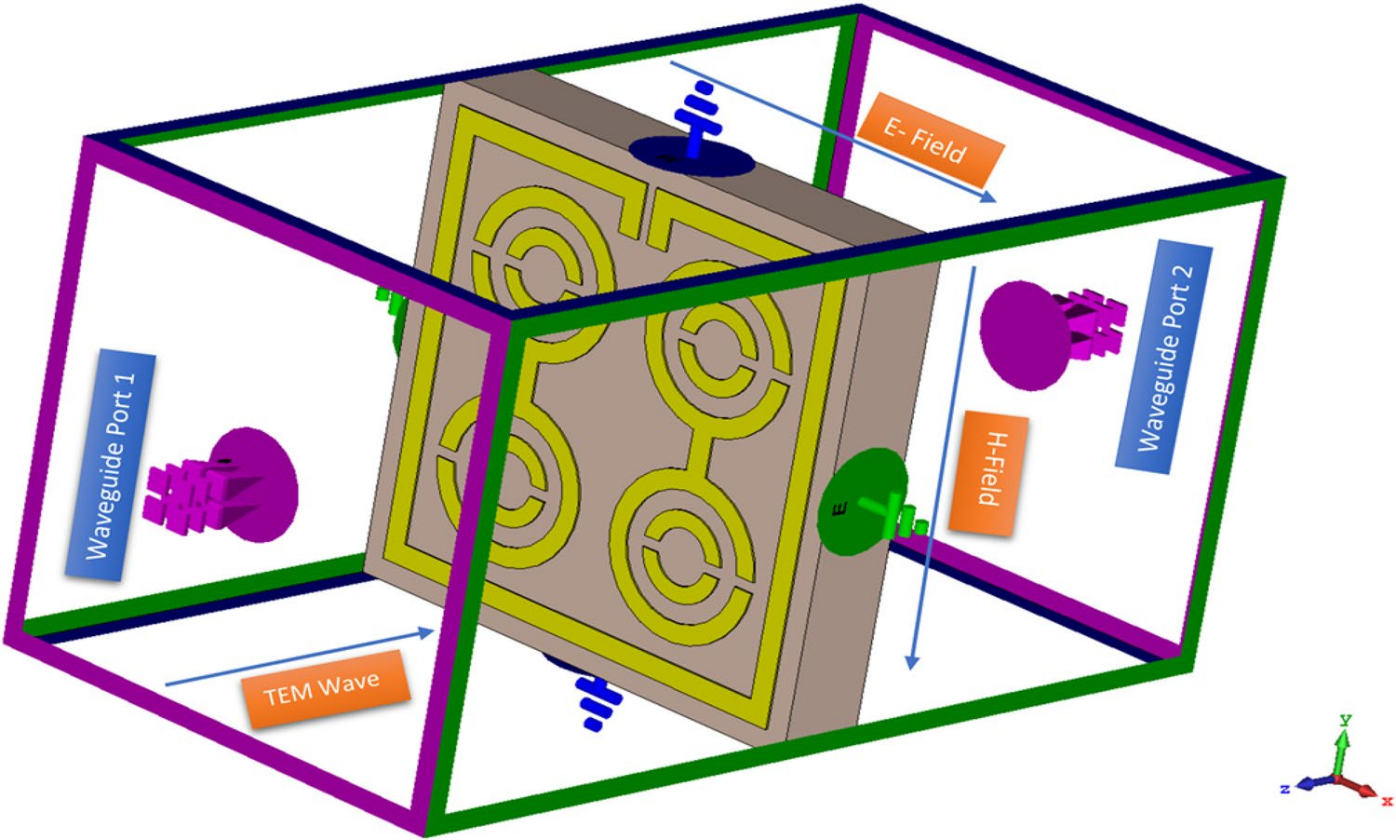
Simulation arrangement of proposed MDD-SRR structure.

To extract effective valid parameters, we employed a Finite Integration Technique (FIT)-based high-frequency electromagnetic simulator CST microwave studio that was operated in a frequency range of 2–18 GHz using a hexahedral mesh. The transverse electromagnetic (TEM) wave propagating along the Z-axis through the proposed metamaterial unit cell and array structure was used to demonstrate the interaction between the fields. As depicted in Fig. 2, the electric field moves along the X-axis, whereas the magnetic field acts on the Y-axis. Whereas, the effective parameters were extracted using the Robust method^41^. Meanwhile, the relationship between the impedance, refractive index, reflection coefficient (|S11|) and transmission coefficient (|S21|) were expressed using the following equations:

1 $$\left|{S}_{11}\right|= \frac{{R}_{01}(1-{e}^{i2n{k}_{0}d})}{1-{{R}^{2}}_{01}{e}^{i2n{k}_{0}d}},$$

2 $$\left|{S}_{21}\right|= \frac{(1-{{R}^{2}}_{01}){e}^{i2n{k}_{0}d}}{1-{{R}^{2}}_{01}{e}^{i2n{k}_{0}d}},$$

where $${R}_{01}= \frac{z-1}{z+1}$$

By inverting Eqs. (1) and (2), the impedance z can be obtained by

3 $$\mathrm{Impedance }z= \pm \sqrt{\frac{{(1+{S}_{11})}^{2}-{({S}_{21})}^{2}}{{(1-{S}_{11})}^{2}-{({S}_{21})}^{2}}.}$$

$${e}^{in{k}_{0}d}=X\pm i\sqrt{1-{X}^{2}}$$ , where $$X= \frac{1}{2 {S}_{21}(1-{S}_{11}^{2}+{S}_{21}^{2})}.$$

The refractive index can be determined by the value of ${e}^{in{k}_{0}d}$,

4 $$\mathrm{Refractive index},n= \frac{1}{kd}\bigg\{\left[{[\mathrm{ln}({e}^{in{k}_{0}d})]}^{"}+2m\pi \right]-i{\left[\mathrm{ln}\left({e}^{in{k}_{0}d}\right)\right]}^{^{\prime}},$$

where (.)′ and (.)″ indicate real and imaginary parts, respectively. Permittivity and permeability can be resolved by impedance (z) and refractive index (n). Also, permittivity (ε) = n/z and permeability (μ) = nz.

The NRW approach^42,43^ was used to further verify the outcome obtained from the CST. The extracted effective medium properties using NRW can be represented using the following equations.:

5 $$ {\text{V}}_{{1}} = \, \left| {{\text{S}}_{{{21}}} } \right| \, + \, \left| {{\text{S}}_{{{11}}} } \right|, $$

6 $$ {\text{V}}_{{2}} = \, \left| {{\text{S}}_{{{21}}} } \right| \, - \, \left| {{\text{S}}_{{{11}}} } \right|. $$

Using Eqs. (5) and (6),

7 $$\left|{S}_{11}\right|= \frac{\left(1-{\Gamma }^{2}\right)z}{1-{\Gamma }^{2}{z}^{2}},$$

8 $$\left|{S}_{21}\right|= \frac{\left(1-{z}^{2}\right)\Gamma }{1-{\Gamma }^{2}{z}^{2}},$$

where, $$\Gamma =X\pm \sqrt{{X}^{2}-1}$$ if $$X= \frac{1-{V}_{1} {V}_{2}}{{V}_{1}-{V}_{2}}.$$

The effective permittivity (ɛ_r_) is presented by

9 $${\varepsilon }_{r}\sim \frac{2}{j{k}_{0}d}\times \frac{(1-{V}_{1})}{(1+{V}_{1})}.$$

The effective permeability (μ_r_) can be represented by,

10 $${\mu }_{r}\sim \frac{2}{j{k}_{0}d}\times \frac{(1-{V}_{2})}{(1+{V}_{2})}.$$

The refractive index (η_r_) can be obtained through,

11 $${\eta }_{r}= \frac{2}{j{k}_{0}d} \times \sqrt{\left\{\frac{{({S}_{21}-1)}^{2}-{{S}_{11}}^{2}}{{({S}_{21}+1)}^{2}-{{S}_{11}}^{2}}\right\},}$$

where $${k}_{0}= \frac{2\pi f}{c}.$$

The MATLAB code was used to extract the effective medium parameters using the above equations.

**Figure 3:**
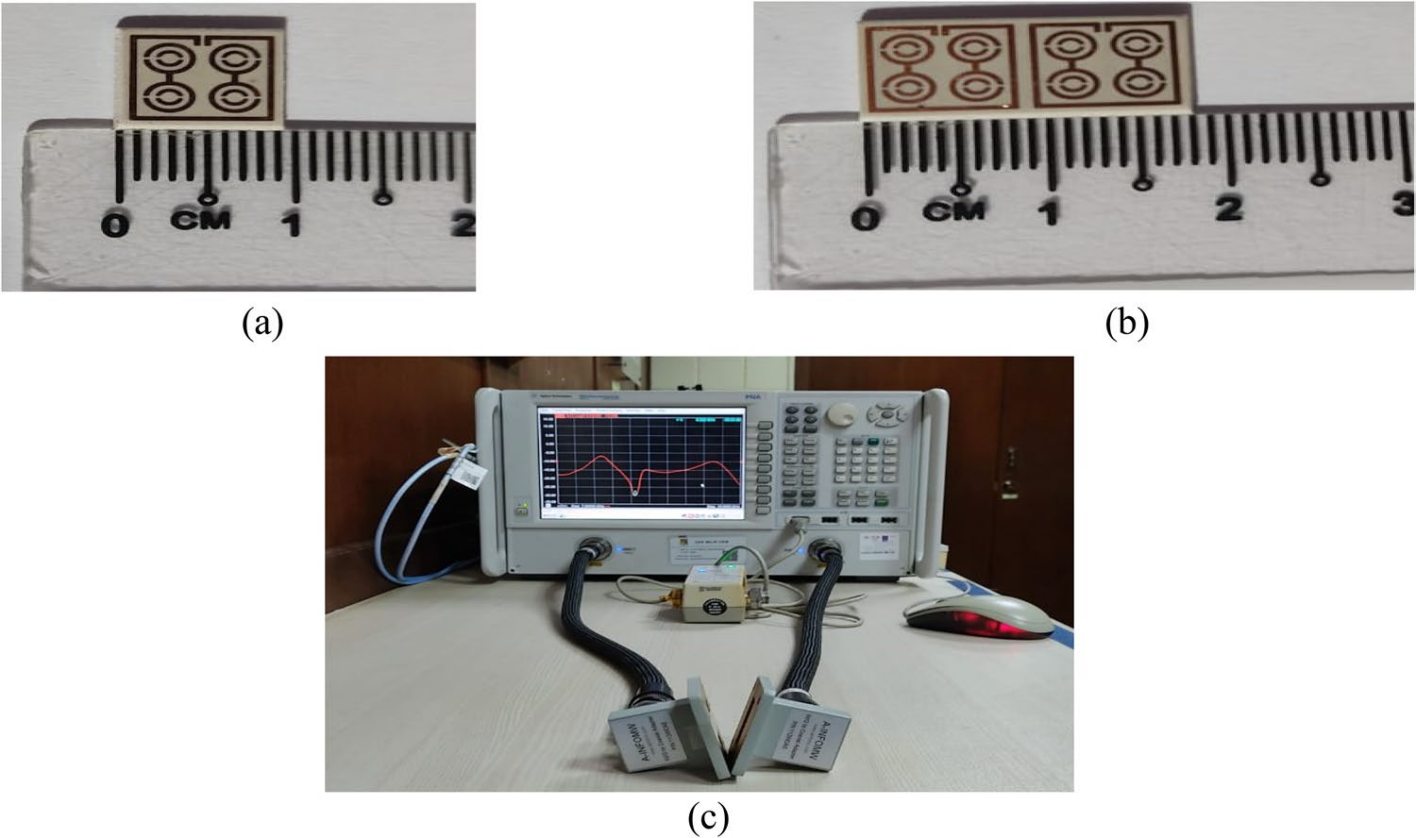
Design prototype of (**a**) unit cell (**b**) 1 × 2 array (**c**) experimental setup to measure the S-parameters using waveguide.

In order to measure the proposed metamaterial structure for validation of the simulated results, an Agilent N5227 PNA Microwave Network Analyzer was used to extract the transmission coefficient (|S21|). The metamaterial structure prototype is placed in between the two waveguide ports such as A-INFOMW W/G to Coaxial Adapter P/N:340WCAS (2.20–3.30 GHz), A-INFOMW W/G to Coaxial Adapter P/N:112WCAS (7.05–10.0 GHz) and A-INFOMW W/G to Coaxial Adapter P/N:75WCAS (10–15 GHz). The Agilent N4694-60001 Electrical Calibration Kit is used to calibrate the microwave network analyzer. Figure 3a,b indicates the prototype of the fabricated metamaterial unit cell and array, whereas Fig. 3c shows the experimental setup.

### Equivalent circuit model of the proposed MDD-SRR metamaterial unit cell

The proposed metamaterial unit cell exhibited quintuple resonances due to the formation of an LC resonance circuit. The resonance frequency of this circuit was measured using Eq. (12) ^44^.

12 $$f=\frac{1}{2\pi \sqrt{LC}},$$

where L is the inductance and C is the capacitance of the metamaterial structure. In the metamaterial unit cell, the inductance was constructed by the metal strip and formed the capacitance by a split gap of the ring. The capacitance is represented by:

13 $$C={\epsilon }_{0}{\epsilon }_{r}\frac{A}{d},$$

where $\epsilon$_0_ is the permittivity in free space, $\epsilon$_r_ is the relative permittivity, A is the area of split conducting strip, and d is the split distance.

The whole inductance was measured based on the transmission line principle^45^:

14 $$L=0.01\times {\mu }_{0}\left\{\frac{2{(d+g+h)}^{2}}{{(2w+g+h)}^{2}}+\frac{\sqrt{{(2w+g+h)}^{2}+{l}^{2}}}{(d+g+h)}\right\}t.$$

Also, the whole capacitance was measured using:

15 $$C={\epsilon }_{0}\left[\frac{2w+g+h}{2\pi {(d+h)}^{2}}\mathrm{ln}\left\{\frac{2(d+g+h)}{(a-l)}\right\}\right]t,$$

where $\epsilon$_0_ = 8.854 × 10^–12^ F/m, μ_0_ = 4π × 10^–7^ H/m, w is the microstrip line width, h is the substrate thickness, t is the microstrip line thickness, and *l* is the length.

**Figure 4:**
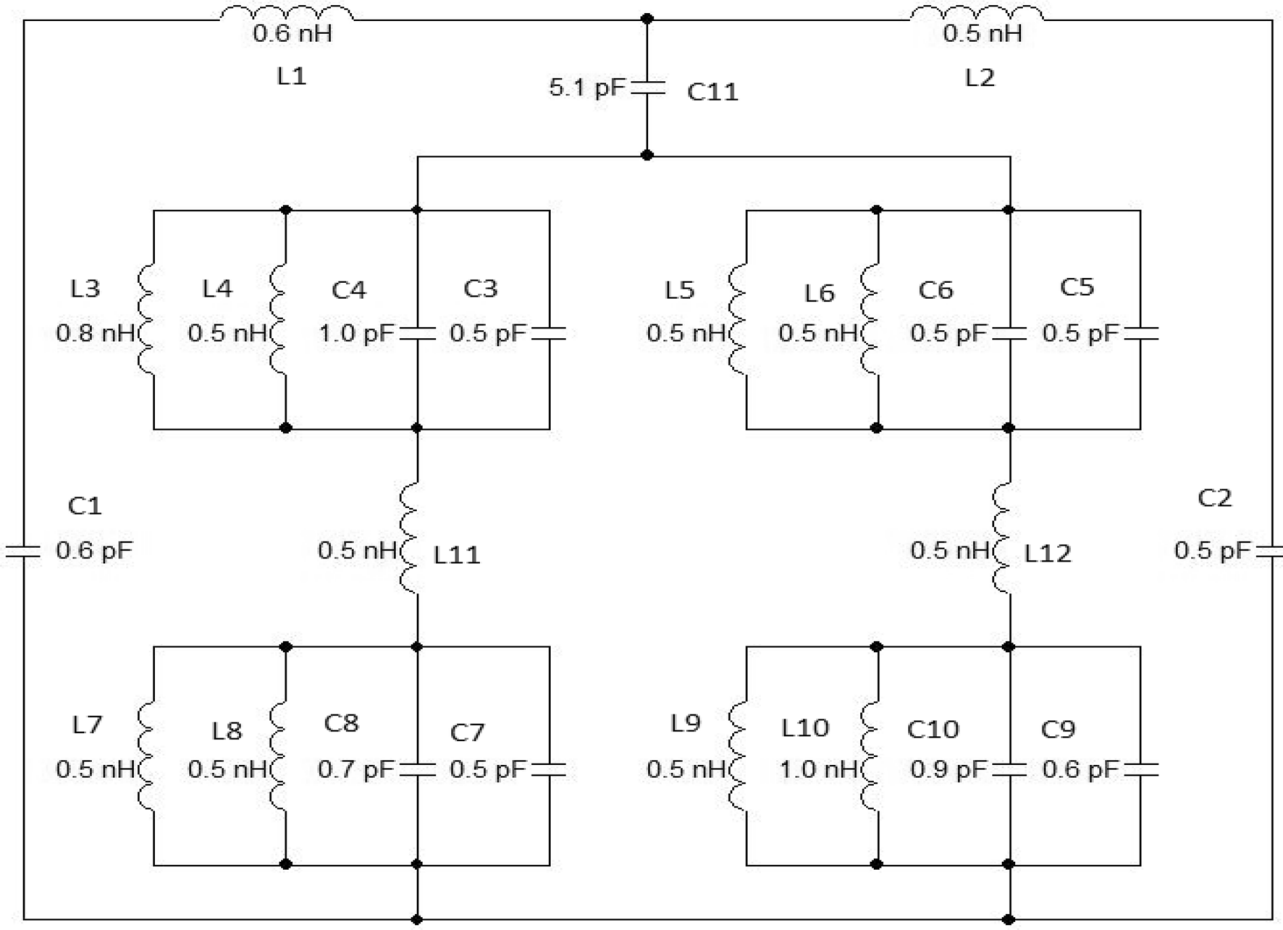
Equivalent circuit of the proposed unit cell.

**Figure 5:**
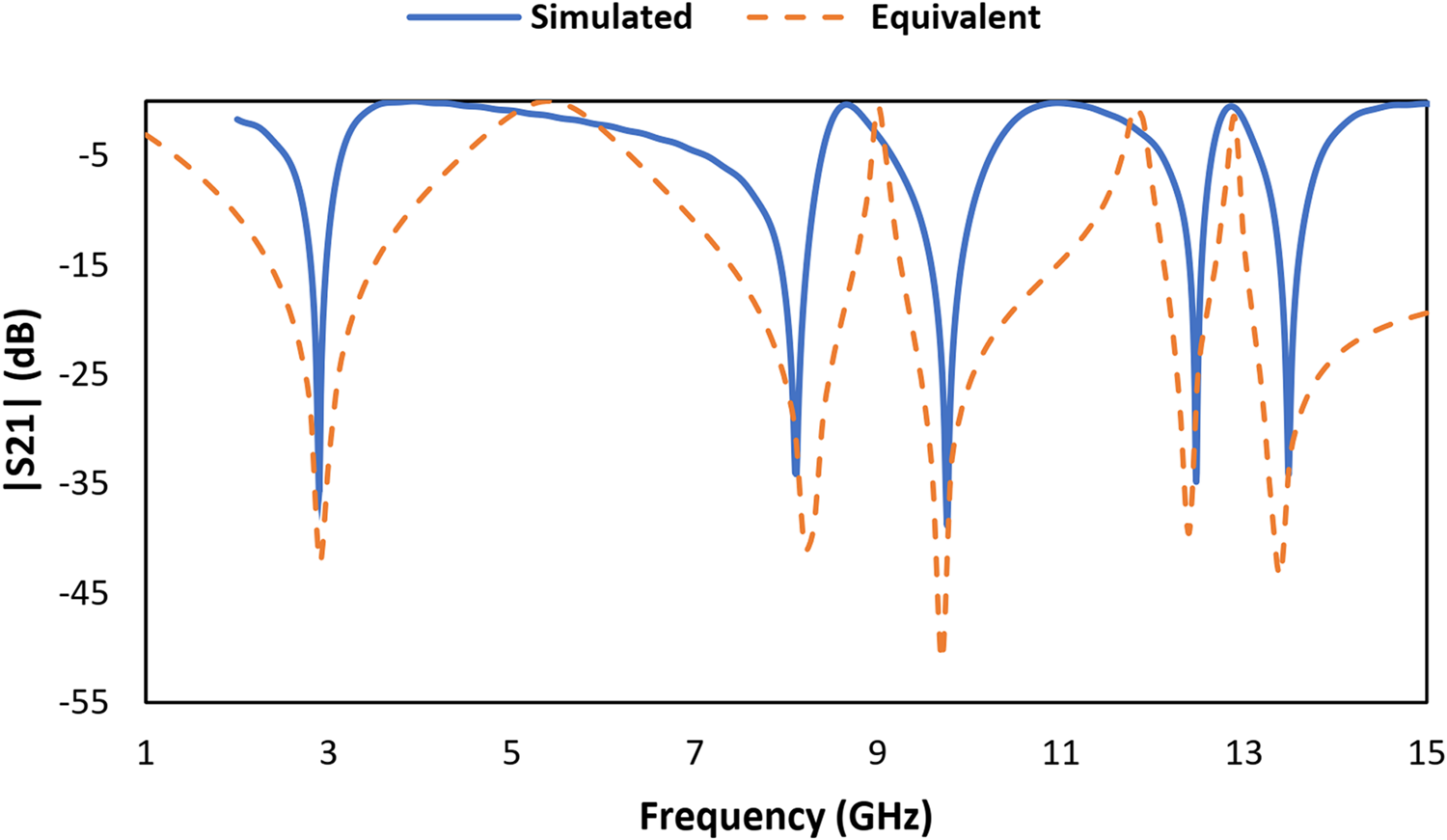
Simulated and equivalent results of |S21| of the proposed unit cell.

The approximated equivalent circuit of the proposed unit cell of an MDD-SRR-based metamaterial using passive elements is depicted in Fig. 4, responsible for creating resonance in the construction of the metamaterial. The equivalent elements of the outer square ring contain two resonance frequencies which were generated by L1, L2 and C1, C2. The half dumbbell-shaped ring contains two inductance and two capacitance values. Whereas, the ring connector indicates the inductance value. One form of dumbbell shape is responsible for two resonance frequencies. The values of inductance and capacitance were optimised in this equivalent circuit using an ADS simulator. In this circuit, L1 and C1 are responsible for 2.896 GHz resonance frequency, meanwhile, the combinations of L2 and C2, L3 and C3, L4 and C4, L5 and C5, L6 and C6, L7 and C7, L8 and C8, L9 and C9, L10 and C10, L11, L12 and C11 are respectively responsible for 8.11 GHz, 9.76 GHz, 12.48 GHz, and 13.49 GHz, respectively. Simulation results of equivalent circuits were also accepted to compare and validate the simulation results achieved through CST (Fig. 5).

### Electromagnetic field analysis of proposed unit cell

In metallic conductors, the surface current represents the real electric current produced by an applied time-varying EM field. EM fields are generated by time-varying electric charges in space that indicate the relationship between electric and magnetic fields. Maxwell and the conventional law contributed several equations to explain the relationships among the material, E-field and H-field, through the following Eqs. (16–17):

16 $$\nabla \times H=J+ \frac{\partial D}{\partial t} \, \mathrm{and } \, \nabla \times E= -\frac{\partial B}{\partial t},$$

17 $$D\left(t\right)= \varepsilon \left(t\right)\times E\left(t\right) \, \mathrm{ and } \, B\left(t\right)= \mu \left(t\right)\times H\left(t\right),$$

where *E* is time-varying electric intensities, *H* represent magnetic field intensities, *D* is time-varying electric densities, *B* is magnetic flux densities, *ɛ* represents electric permittivity, *μ* is magnetic permeability, *J* is the time-varying electric current density in a medium and $\nabla =\left[\frac{\partial }{\partial x},\frac{\partial }{\partial y} , \frac{\partial }{\partial z}\right]$.

**Figure 6:**
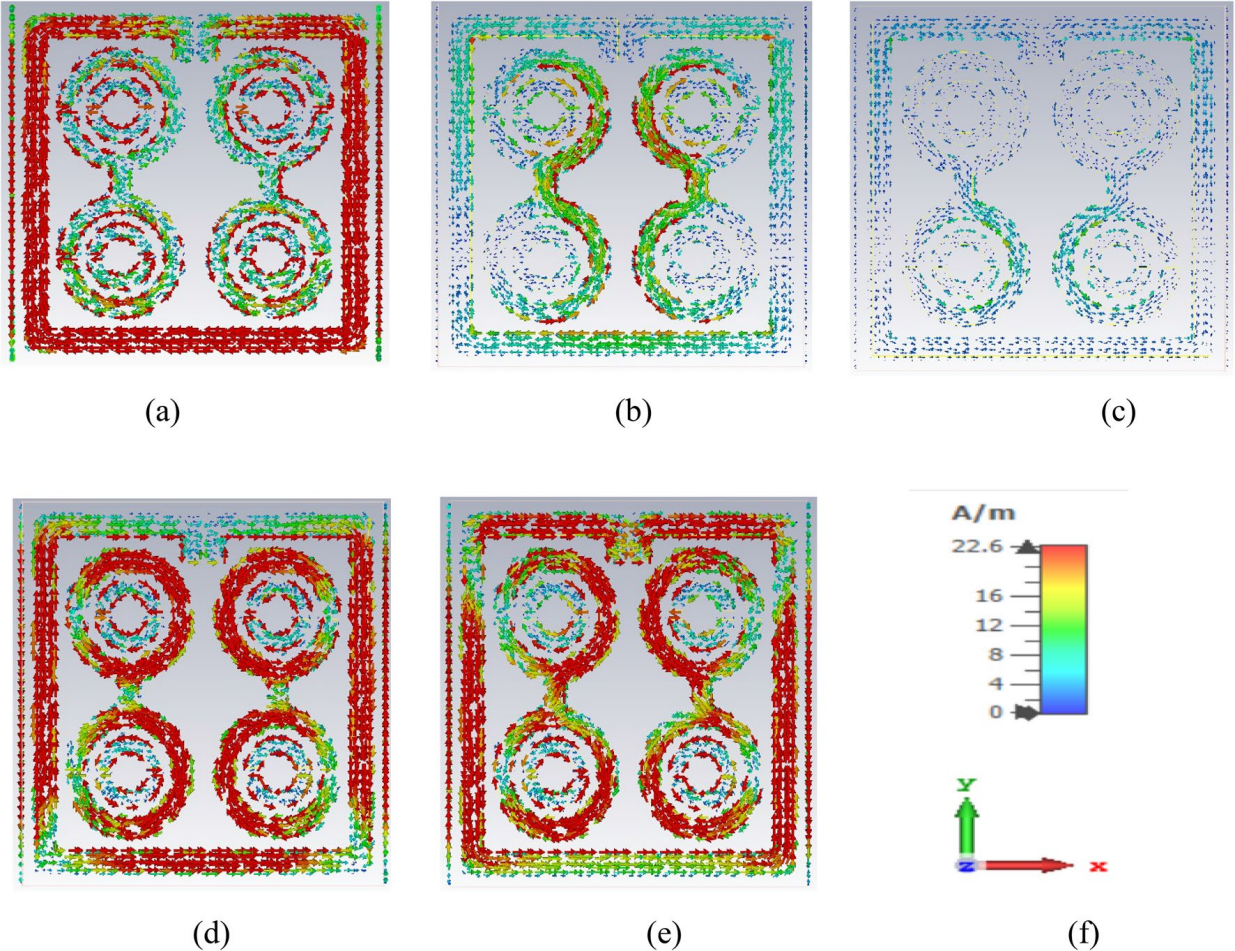
Surface current distribution of unit cell at (**a**) 2.896 GHz (**b**) 8.11 GHz (**c**) 9.76 GHz (**d**) 12.48 GHz (**e**) 13.49 GHz and (**f**) axis.

The electromagnetic field analysis of the proposed unit cell can be clarified using the Eqs. (16–17). Figure 6 illustrates the surface current distribution of the proposed unit cell at the frequencies of 2.896 GHz, 8.11 GHz, 9.76 GHz, 12.48 GHz and 13.49 GHz. Based on Fig. 6a, the outer square ring provided a strong surface current because it provided a low impedance path at 2.896 GHz. Moreover, the two dumbbell-shaped rings generated a little bit of current. At 8.11 GHz, as indicated in Fig. 6b, the surface current was distributed uniformly. The lower current densities were observed in all portion of the cells except the inner part of the dumbbell-shaped upper circle. The two vertical edges and the upper horizontal edge of the outer square ring also contributed less to the surface current flow due to the greater capacitive response for the split gap. Meanwhile, the current density in the upper circle of the two dumbbell shapes increased rapidly at 9.76 GHz. According to Fig. 6c, the distributed surface current was observed on three sides of the outer square ring at this frequency. The entire metamaterial unit cell except for the horizontal sides of the outer square ring contributed most of the surface current at the resonance frequency of 12.48 GHz (Fig. 6d). Whereas, at 13.49 GHz, a lower current density was recorded throughout the unit cell, as a high frequency causes an increase in impedance (Fig. 6e).

**Figure 7:**
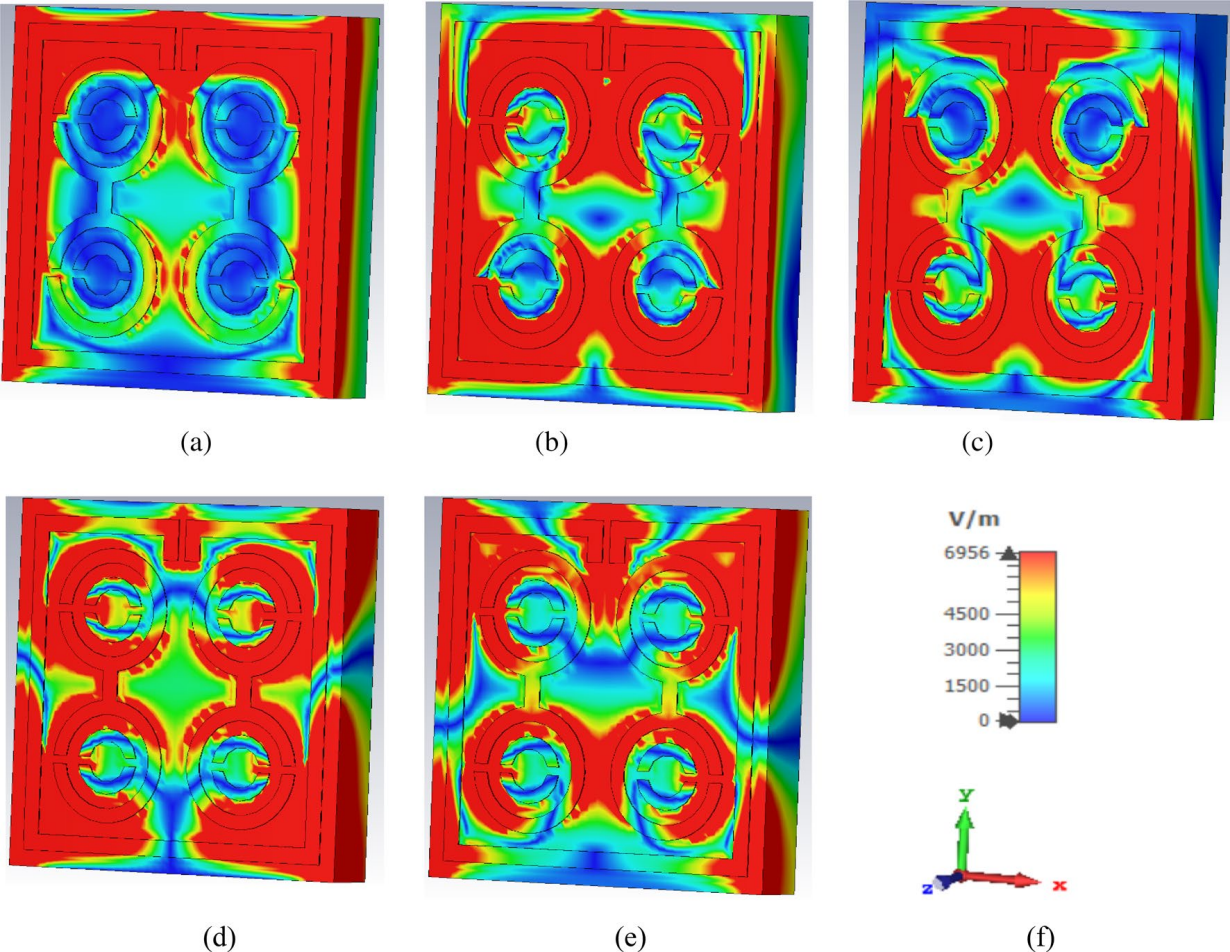
E-field distribution of unit cell at (**a**) 2.896 GHz (**b**) 8.11 GHz (**c**) 9.76 GHz (**d**) 12.48 GHz (**e**) 13.49 GHz and (**f**) axis.

**Figure 8:**
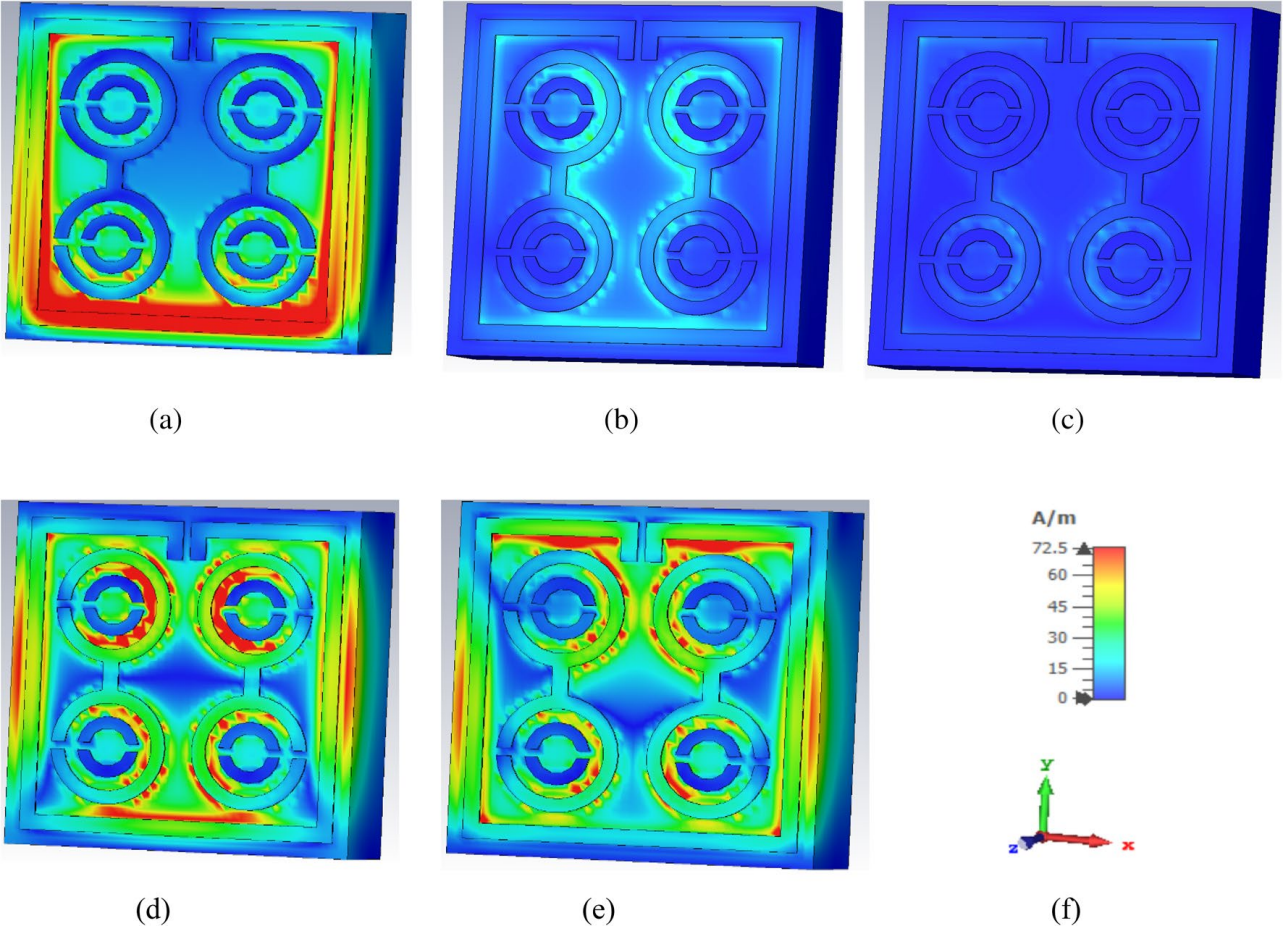
H-field distribution of unit cell at (**a**) 2.896 GHz (**b**) 8.11 GHz (**c**) 9.76 GHz (**d**) 12.48 GHz (**e**) 13.49 GHz and (**f**) axis.

Based on the results, a good relationship between the surface current of the unit cell and the H-field was observed, confirmed by Ampere’s law. Higher current increased the intensity of the H-field, hence, was noticeable in the H-field (Fig. 8). From the study of the surface current in Fig. 6a, it was apparent that the surface current density in the outer square ring was comparatively higher than that of the other parts. Therefore, the H-field was strong in places with high current density (Fig. 8a). Meanwhile, the upper part of the outer square ring manifested a very low current flow due to the split gap, resulting in the H-field being close to zero. On the other hand, the strength of the E-field gradually increased at the point of splits because the split in the outer square ring forms the capacitor creating an additional electric field, as indicated in Fig. 7a. According to Maxwell Law, the greater the amount of H-field in the outer square ring, the greater the amount of E-field on the opposite side of the outer square ring, verified in Figs. 7 and 8. In short, the strength of the E-field increases if the rate of change of the H-field is negative. Hence, it can be concluded that E-field and H-field models follow the relationships depicted in Eqs. (16) and (17).

## Results and discussion

**Figure 9:**
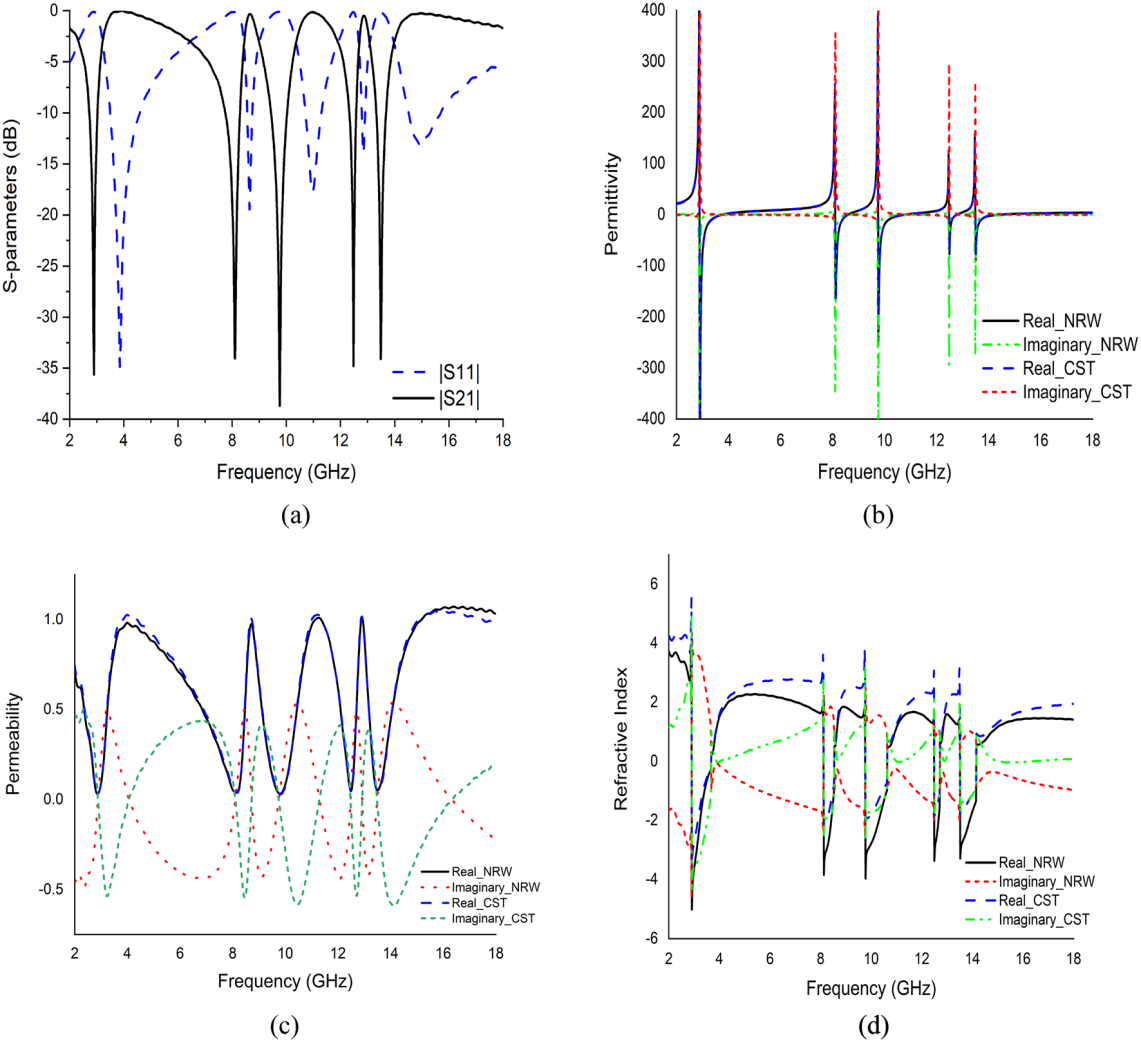
Simulated results of the proposed unit cell (**a**) Scattering parameters, (**b**) Permittivity graph, (**c**) Permeability graph, and (**d**) Refractive index graph.

The popular CST simulator acquired the S-parameters of the proposed MDD-SRR based unit cell, the reflection coefficient (|S11|) and transmission coefficient (|S21|) as depicted in Fig. 9a. The unit cell offered quintuple resonances with frequencies of 2.896 GHz, 8.11 GHz, 9.76 GHz, 12.48 GHz and 13.49 GHz with an amplitude of − 35.4 dB, − 33.4 dB, − 38.4 dB, − 34.7 dB and − 33.6 dB, respectively. Having said that, bandwidth is an important component of satellite communications. The proposed unit cell in this study as depicted in Fig. 9a displays excellent bandwidth with the value of transmission coefficient of less than − 10 dB in the ranges of 2.73–3.03 GHz, 7.75–8.29 GHz, 9.44–10.03 GHz, 12.33–12.57 GHz and 13.33–13.68 GHz. The reflection coefficient (|S11|) of the unit cell exhibited five resonances at the frequencies of 3.85 GHz, 8.64 GHz, 10.99 GHz, 12.86 GHz and 14.93 GHz with an amplitude of − 34.36 dB, − 19.41 dB, − 17.73 dB, − 13.75 dB and − 13.15 dB, respectively. Figure 9a demonstrates that each resonance frequency of the reflection coefficient is followed by a maximum transmission coefficient. The permittivity and permeability along with boundary condition where the magnetic field and electric field is travelled Y-axis and X-axis in a medium determine uniquely the response of the artificial metamaterial unit cell design to an incoming time-varying electromagnetic wave which can be influenced by the change in the geometric shape of the proposed unit cell.

There are several methods like the Drude method, TR-method, NRW method and Robust method used to obtain the effective parameter of a metamaterial. In this study, the NRW approach in MATLAB programming and the Robust retrieval method in CST was applied to extract the effective medium parameter response from S-parameters, as indicated in Fig. 9b,c. The recorded permittivity was negative at each resonance frequency with an amplitude of − 642, − 137, − 195, − 20 and − 60, respectively for both NRW and Robust methods, while the values of permeability measured were close to zero at each resonance with the amplitude of 0.034, 0.040, 0.035, 0.047 and 0.050, respectively. Therefore, the proposed MDD-SRR based unit cell exhibited the ENG property, that can be used to increase the antenna bandwidth, microwave filter design, etc.^46^. The imaginary part of the permeability shows negative at every resonance frequency which is shown in Fig. 9c. Meanwhile, Fig. 9d indicates that the refractive index was negative at frequencies ranging between 2.912–3.664 GHz, 8.128–8.528 GHz, 9.776–10.624 GHz, 12.496–12.704 GHz and 13.52–14.144 GHz with an amplitude of − 3.5, − 2.16, − 1.86, − 1.85 and − 1.82, respectively. Each of the negative maximum value of the refractive index occurred near the resonance frequency.

The effective medium ratio (EMR) determined the goodness of the metamaterial unit cell design control for satellite communication. As indicated in Eq. (18), the EMR is calculated as the wavelength to dimension ratio of the metamaterial unit cell design. Negative permittivity is conceivable with the proposed metamaterial design if the EMR value is more than 4, which is an ideal number. The proposed metamaterial unit cell has an EMR of 11.51.

18 $$\mathrm{EMR}=\frac{\mathrm{Wavelength},\uplambda }{\mathrm{Unit}  \, \mathrm{cell}  \,  \mathrm{length},\mathrm{ L}}.$$

**Figure 10:**
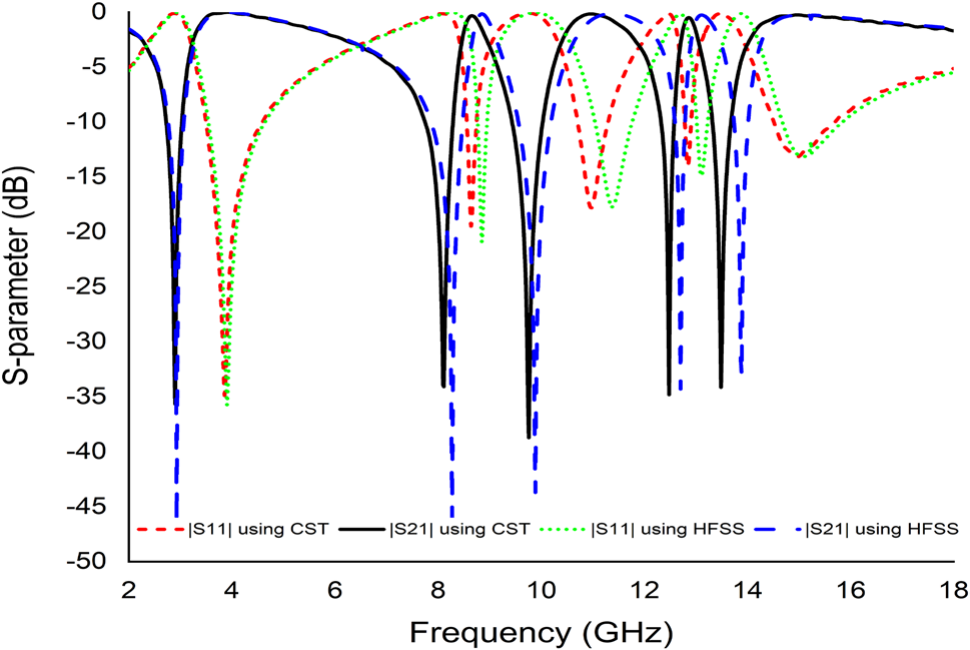
S-parameter results of MDD-SRR unit cell using CST and HFSS simulator.

Similarly, HFSS was adopted to compare and justify the simulated results achieved using the CST simulator as illustrated in Fig. 10. The two simulators are considered in this study because each employs various techniques. CST operates according to the Finite Integral Technique (FIT) approach, while HFSS uses the Finite Element Method (FEM). Simulation results using HFSS have also been accepted to compare and validate the simulation results achieved with Computer Simulation Technology (CST) and are introduced in Fig. 10. The proposed unit cell displayed five resonances in both simulators with slight fluctuations in the last four resonance frequencies.

**Figure 11:**
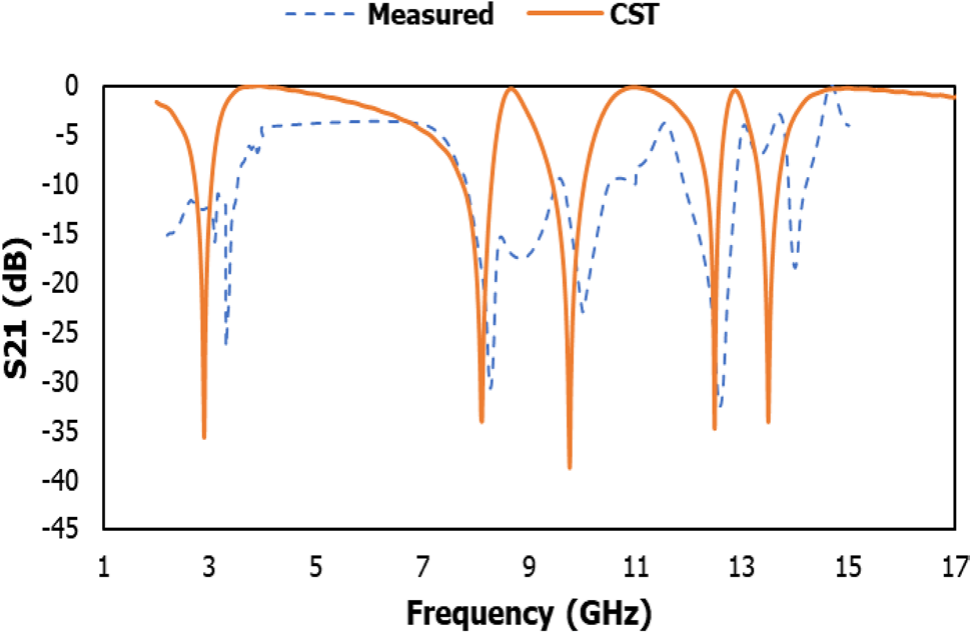
The transmission coefficient results of MDD-SRR unit cell using CST and experimental.

The simulation and experimental results of the proposed metamaterial unit cell were in close agreement with each other for validation purposes, as shown in Fig. 11. The measured resonance frequencies of the metamaterial unit cell structure were 3.30 GHz, 8.26 GHz, 9.99 GHz, 12.56 GHz and 13.97 GHz, including the S, X and Ku band using Agilent N5227 PNA Microwave Network Analyzer. Because of the small manufacturing and calibration error and also the mutual resonance effect of the two waveguides, the measurement result remains only slightly inconsistent with the simulation result.

### Parametric studies

#### The effect of changing the various design steps

**Figure 12:**
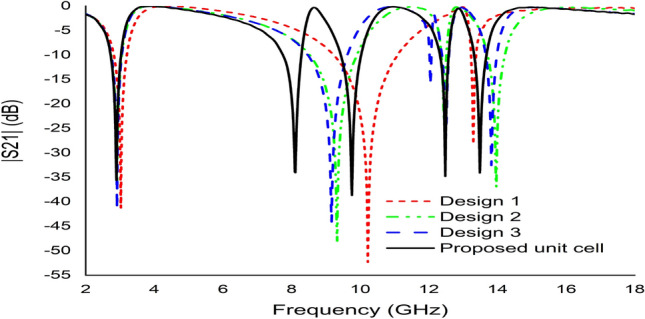
The transmission coefficient graph based on various design steps.

**Figure 13:**
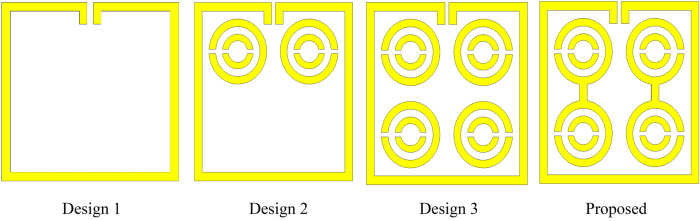
Various step to finalize the MDD-SRR structure.

**Figure 14:**
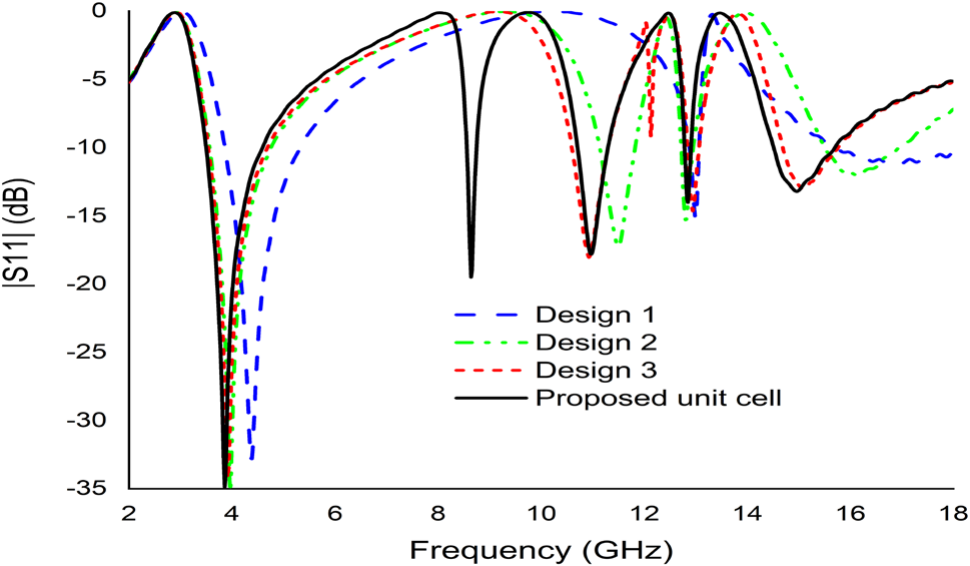
The reflection coefficient graph based on different design steps.

The metamaterial unit cell was developed based on a trial-and-error method to obtain the maximum number of resonances with negative permittivity and excellent EMR. Different designs were tested one after another until the desired property was achieved using the iterative method. The unit cell design began with the introduction of a square split ring resonator with a size of 9 mm on each side with an upper side split gap of 0.2 mm. This design exhibited three resonant frequencies that covered the S-, X-, and Ku-bands as illustrated in Fig. 12. In the next step, two circular rings with three separate split gaps were added to the square ring as indicated in Fig. 13. According to Fig. 12, an additional resonance at a frequency of 13.97 GHz was created by inserting it into a square ring. Two more rings with a split gap were installed vertically in Fig. 13 indicated additional resonance at 12.05 GHz with − 15 dB. The design was finalised by connecting the circular split rings vertically to create two dumbbell shape, as illustrated in Fig. 13. The design represented five main resonances around 2.896 GHz, 8.11 GHz, 9.76 GHz, 12.48 GHz and 13.49 GHz, covering the frequency bands of S-, X- and Ku-bands satellite communications. Furthermore, the reflection coefficient of all the designs indicated the same resonant frequencies as the transmission coefficient as indicated in Fig. 14.

#### The effects of modifying the split gap of rings

**Figure 15:**
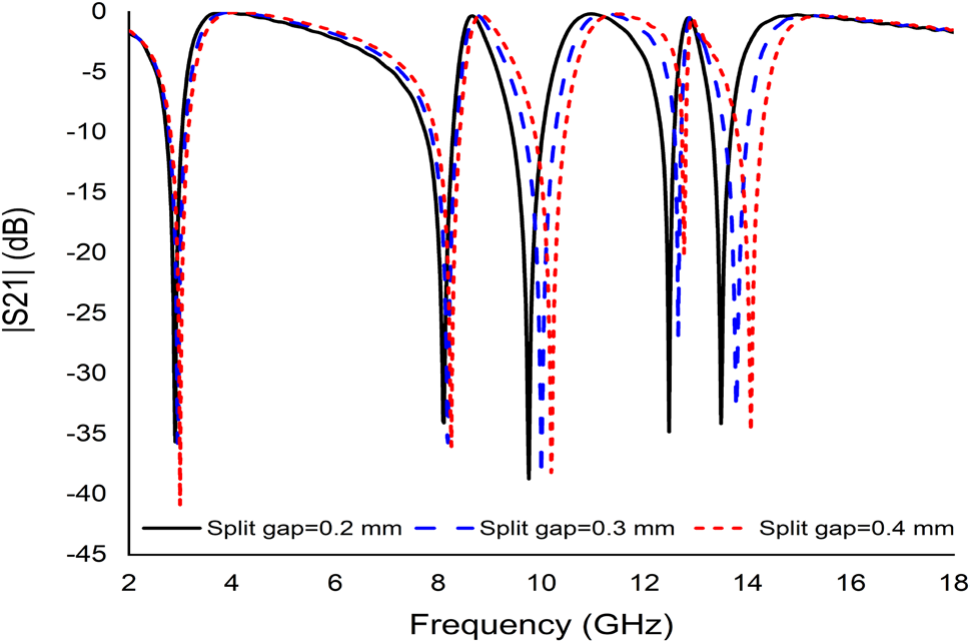
The transmission coefficient graph based on various split gap.

In this study, the effects of modifying the split gap of the whole ring on the proposed unit cell were also investigated. The |S21| of the metamaterial unit cell for different separate split gaps was introduced in Fig. 15. The resonance frequency of the unit cell depends on the electrical length of the copper and the split gap of the ring. As the split gap of the ring increases, the capacitance value decreases, affecting the resonant frequency towards a higher value (Fig. 15). The initial design depicted a whole split gap of the unit cell of 4 mm exhibiting the quintuple resonance frequency. The unit cell split gap was gradually reduced to 2 mm with a step size of 1 mm. The value of the resonance frequency was also observed to have reduced. Having said that, a 2 mm split gap also yielded the quintuple resonance frequency, but the value was reduced compared to others, indicating that the capacitive reactance is inversely proportional to frequency.

#### The effects of changing the substrate properties

**Figure 16:**
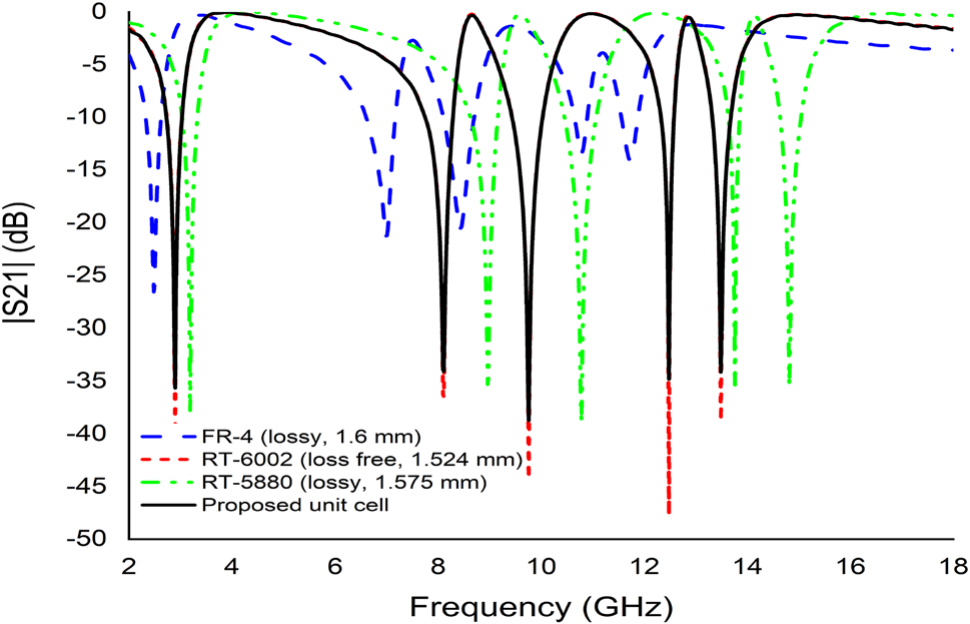
The transmission coefficient graph for different substrate.

The study also examined the effects of changing the substrate material of the unit cell. This analysis used three Rogers variants with the most commonly used FR-4 materials. The first study used the FR-4 (lossy) with a thickness of 1.6 mm, electric permittivity of 4.3 and dielectric loss of 0.025. Figure 16 indicates that this material exhibited five resonances frequency with a lower resonance peak and a narrower bandwidth but higher EMR compared to others. Meanwhile, Rogers RT 5880 (lossy) with a thickness of 1.575 mm, electric permittivity of 2.2 and dielectric loss of 0.0009 also exhibits the same response as the previous design, with an acceptable magnitude. However, two more experiments were performed using Rogers RT 6002 (lossy and loss-free) with a thickness of 1.524 mm, electric permittivity of 2.94, the dielectric loss of 0.0012 (lossy). Both substrate materials demonstrated the same frequency band with an acceptable magnitude as depicted in Fig. 16. Comparatively, the resonance peak of Rogers RT 6002 (lossy) was higher compared to the others. Since RT 6002 (lossy) covered maximum bands with acceptable resonance peak and excellent EMR, the proposed metamaterial unit cell was outlined on this material.

#### The effects of changing the EM field propagation

**Figure 17:**
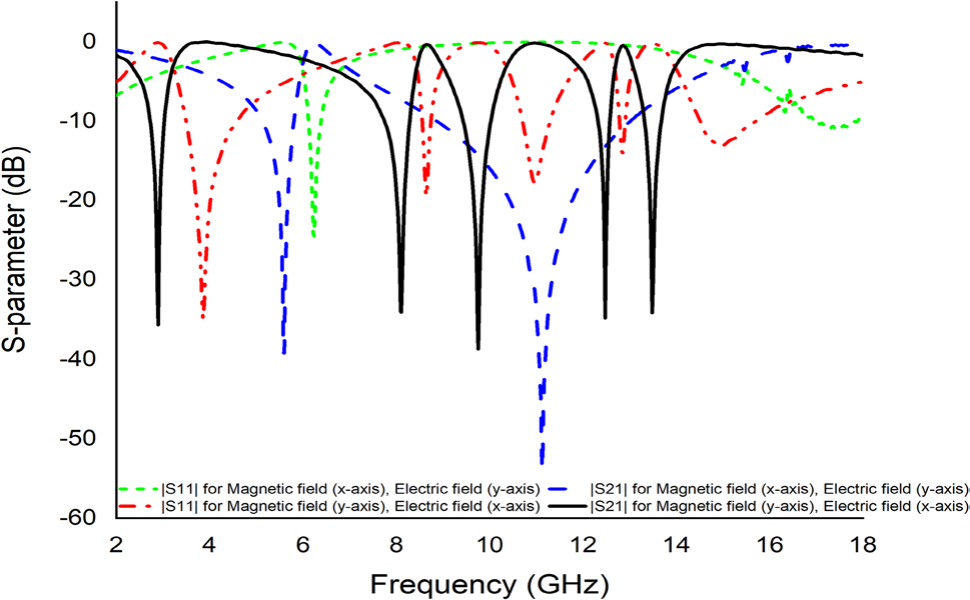
S-parameter for different orientation based on propagation of electromagnetic field.

**Figure 18:**
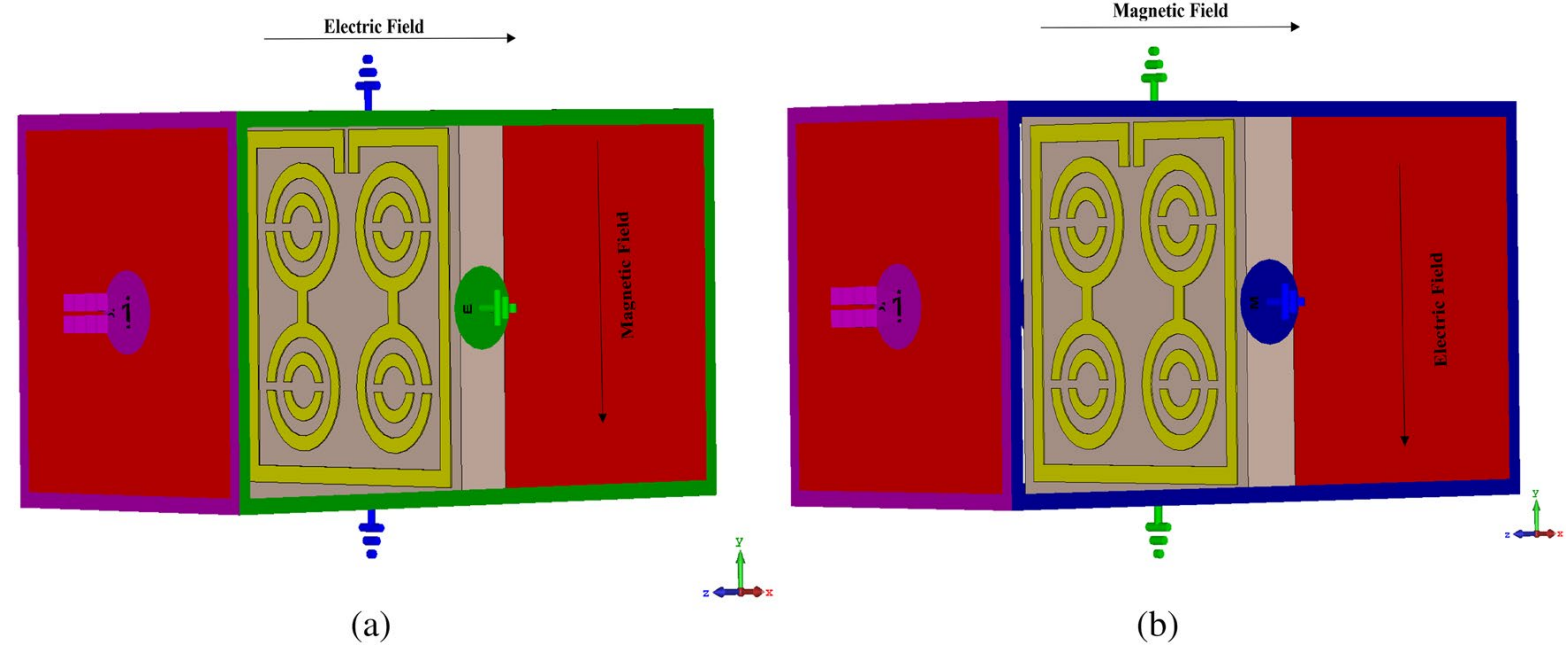
Different orientation of electromagnetic field propagation (**a**) Ex-Hy and (**b**) Ey-Hx.

The simulation setup can be represented based on the different orientations of EM propagation in Fig. 18. An EM field was propagated along the Z-axis, where electric and magnetic fields were perpendicular to each other. According to Fig. 18a, the E-field operated on the X-axis, while the H-field acted on the Y-axis. In this orientation, the metamaterial unit cell exhibited five resonance frequencies for both |S21| and |S11|, as indicated in Fig. 17. On the other hand, when the E-field acted on the Y-axis and the H-field on the X-axis (Fig. 18b), the metamaterial unit cell of this orientation yielded two resonance frequencies for both |S21| and |S11| (Fig. 17). However, only the first orientation was selected because it matched the purpose of this research.

#### The effects of polarisation and incident angle on metamaterial unit cell

**Figure 19:**
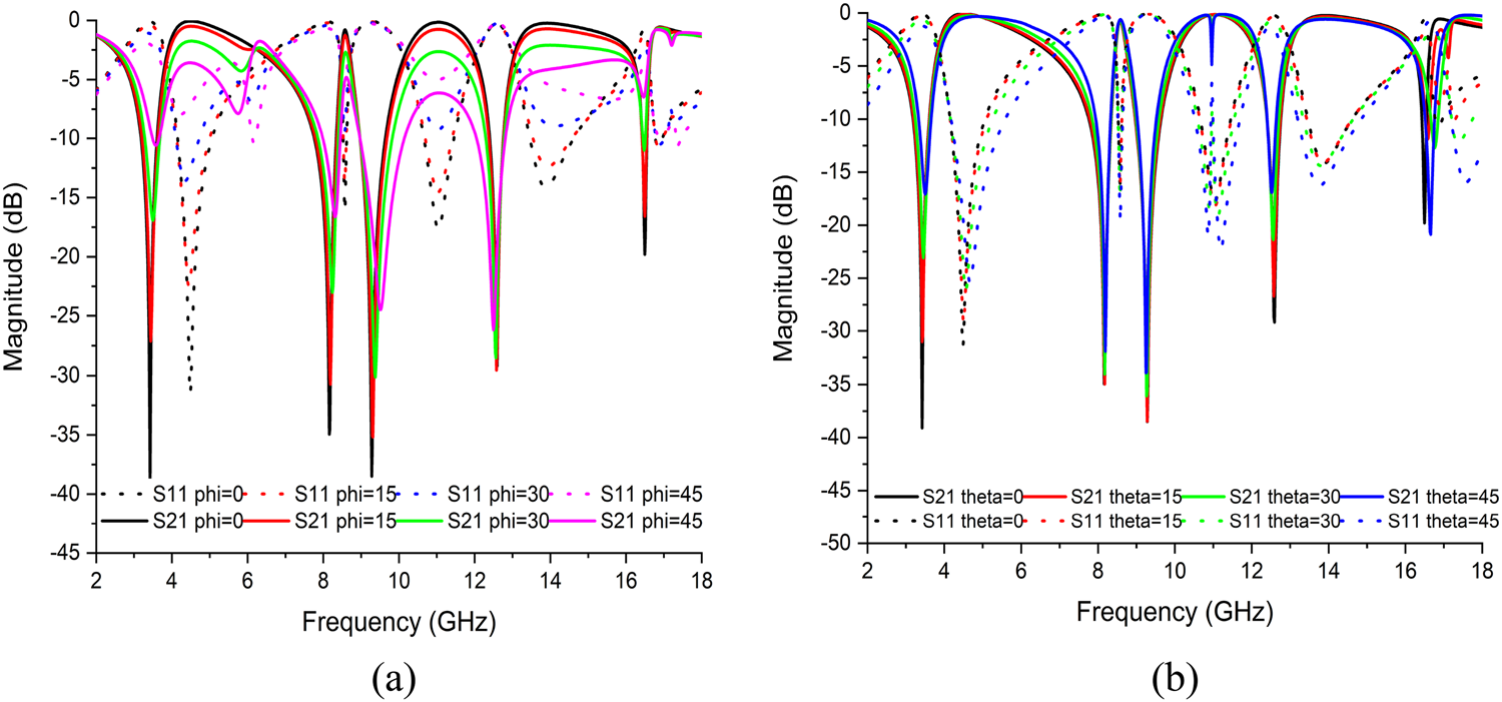
S-parameter for proposed unit cell based on (**a**) polarisation and (**b**) incident angle.

Figure 19a,b illustrate the changes in reflection and transmission coefficients with the frequencies of EM waves for the polarisation (Φ) and incident angle (θ) from 0° to 45°. The numerical simulation was well performed in a high-frequency EM simulator CST microwave studio. Both graphs indicated that when phi or theta increases, the metamaterial revealed incompatible intensities for all resonant frequencies. However, both features produced the same number of resonance frequencies with the metamaterial unit cell. Hence, this metamaterial unit cell enabled polarisation and incident angle insensitivity.

### Array metamaterial results

**Figure 20:**
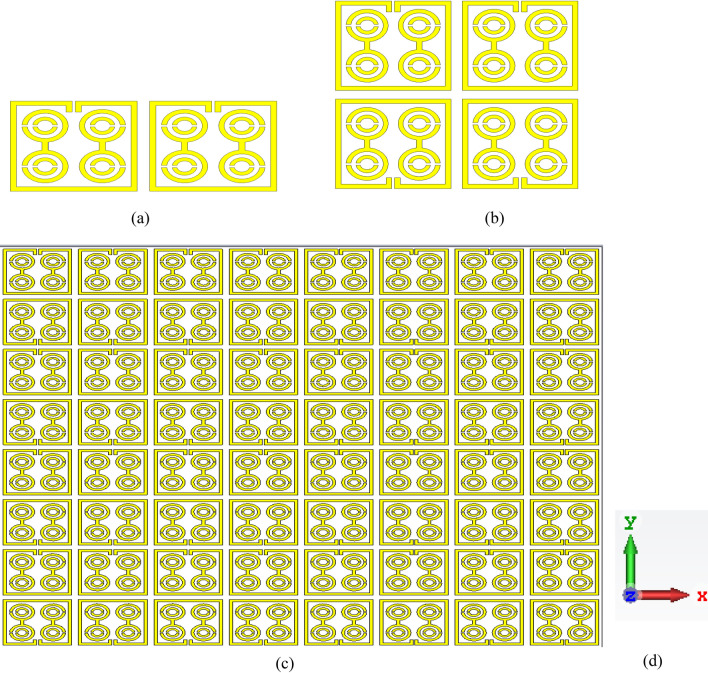
Various types of array (**a**) 1 × 2 (**b**) 2 × 2 (**c**) 8 × 8 (**d**) axis.

**Figure 21:**
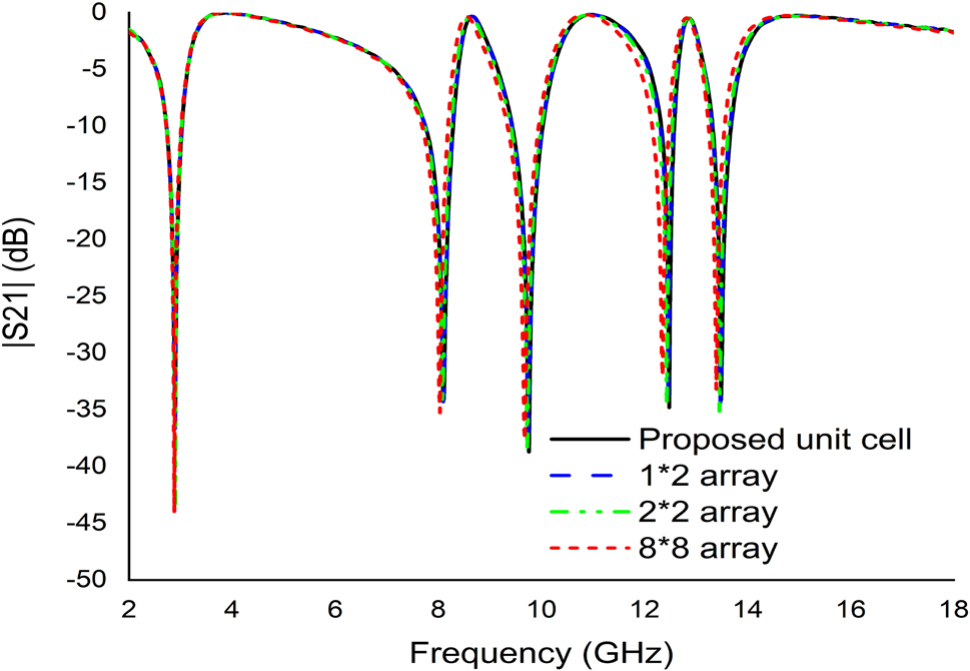
Transmission coefficient results of proposed unit cell, 1 × 2, 2 × 2, and 8 × 8 array.

**Figure 22:**
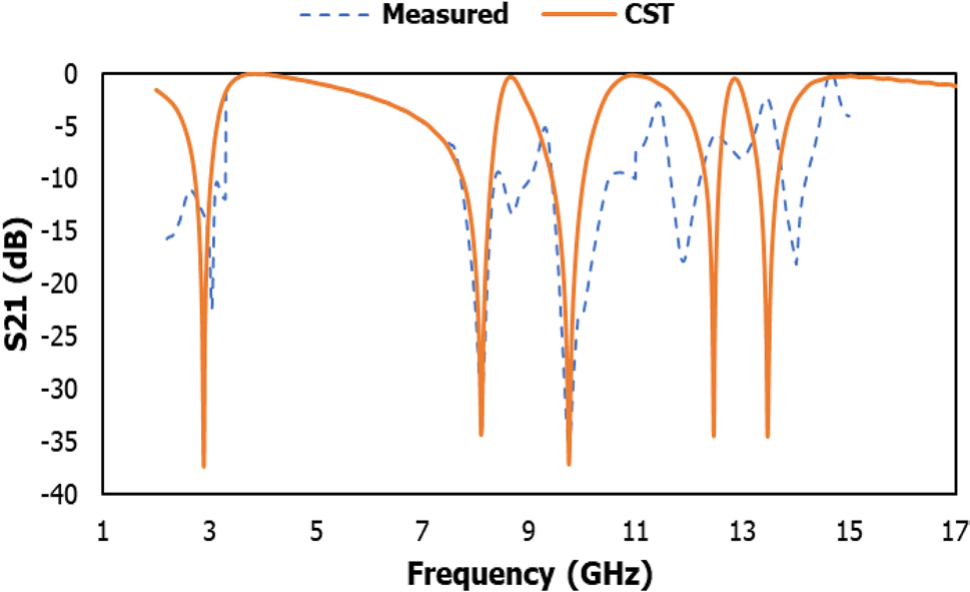
Transmission coefficient results of 1 × 2 array using CST and experimental.

Despite the advantages, metamaterial unit cell arrays need to be inspected because a simple unit cell does not work independently in many practical situations. The different arrangements in an array are described to assess the effects of coupling between the unit cells, as indicated in Fig. 20. The |S21| of the proposed MDD-SRR unit cell 1 × 1 (9 × 9 mm^2^), 1 × 2 (9 × 18 mm^2^), 2 × 2 (18 × 18 mm^2^), 8 × 8 (72 × 72 mm^2^) has been displayed in Fig. 21. Figure 21 illustrates the transmission coefficient of both arrays displaying an equal response since the proposed unit cell exhibited quintuple resonance frequencies. This numerical simulation setup was implemented using the CST microwave simulator. The first resonance peak of the 2 × 2 and 8 × 8 arrays was higher than the others because an array is more modified by harmonics at higher frequencies. The simulated and experimental results of 1 × 2 array have been shown in Fig. 22. It is observed from Fig. 22 that the measured results of 1 × 2 array exhibit same resonances as the simulated results using CST software. The measured resonance frequencies of the 1 × 2 array are 3.05 GHz, 8.09 GHz, 9.75 GHz, 11.90 GHz and 14 GHz, including the S, X and Ku band using two waveguide ports. In addition, the result of the array shows some mismatching at resonance frequencies. But the results show that the array outcome is very similar to the unit cell results showing quintuple resonances. Minor discrepancies may be due to the manufacturing defects and mutual coupling effect.

## Conclusion

This study examined the metamaterial with an MDD-SRR indicating quintuple resonances in S-, X-, and Ku-bands over the operating frequency range of 2–18 GHz. The metamaterial unit cell was created on Rogers RT 6002 with a dimension of 9 × 9 mm^2^. The scattering parameters were measured using CST EM field simulation software, whereas the effective medium parameters were computed using the Robust method and NRW methods. Both methods yielded similar results. The scattering parameters of the metamaterial unit cell was also verified using the Advanced Design System software to optimise the equivalent circuit model and HFSS. The results of the 1 × 2, 2 × 2 and 8 × 8 arrays analysed with the unit cell yielded a good agreement. The simulation result is confirmed by the experimental measurement of proposed unit cell and 1 × 2 array. The measured results show great similarity with the simulated results. The proposed unit cell provided an excellent EMR of 11.51 with proven compactness and criteria met, L < λ/4. The negative permittivity, negative refractive index and near-zero permeability properties proposed by the unit cell can also be used to improve the performance of different microwave frequency devices due to the moderate unit cell size and high EMR. The S- and X-bands are mostly used in airport surveillance radar, weather radar and communication satellites. Meanwhile, the Ku-band is commonly employed in satellite TV networks. Therefore, the metamaterial unit cell design outlined in this work can be effectively used in satellite.
